# Association of Objective and Self‐Reported Sleep Duration With All‐Cause and Cardiovascular Disease Mortality: A Community‐Based Study

**DOI:** 10.1161/JAHA.122.027832

**Published:** 2023-03-09

**Authors:** Binbin Zhao, Yuxuan Meng, Xiaoying Jin, Wenyu Xi, Qingyan Ma, Jian Yang, Xiancang Ma, Bin Yan

**Affiliations:** ^1^ Department of Psychiatry The First Affiliated Hospital of Xi’an Jiaotong University Xi’an China; ^2^ Department of Brain Sciences Imperial College London London UK; ^3^ Department of Clinical Research Center The First Affiliated Hospital of Xi’an Jiaotong University Xi’an China

**Keywords:** all‐cause mortality, cardiovascular disease mortality, objective sleep duration, self‐reported sleep duration, SHHS, Risk Factors, Mortality/Survival

## Abstract

**Background:**

Previous studies found an association between self‐reported sleep duration and mortality. This study aimed to compare the effects of objective and self‐reported sleep duration on all‐cause and cardiovascular disease (CVD) mortality.

**Methods and Results:**

A total of 2341 men and 2686 women (aged 63.9±11.1 years) were selected from the SHHS (Sleep Heart Health Study). Objective sleep duration was acquired using in‐home polysomnography records, and self‐reported sleep duration on weekdays and weekends was based on a sleep habits questionnaire. The sleep duration was categorized as ≤4 hours, 4 to 5 hours, 5 to 6 hours, 6 to 7 hours, 7 to 8 hours, and >8 hours. Multivariable Cox regression analysis was used to investigate the association of objective and self‐reported sleep duration with all‐cause and CVD mortality. During a mean follow‐up period of 11 years, 1172 (23.3%) participants died, including 359 (7.1%) deaths from CVD. All‐cause and CVD mortality rates decreased gradually with increasing objective sleep duration. In multivariable Cox regression analysis, the greatest association for all‐cause and CVD mortality was with an objective sleep duration of 5 hours or shorter. In addition, we found a J‐shaped association of self‐reported sleep duration on both weekdays and weekends with all‐cause and CVD mortality. Self‐reported short (≤4 hours) and long (>8 hours) sleep duration on weekdays and weekends were associated with an increased risk of all‐cause and CVD mortality compared with 7 to 8 hours sleep duration. Furthermore, a weak correlation was observed between objective and self‐reported sleep duration.

**Conclusions:**

This study showed that both objective and self‐reported sleep duration were associated with all‐cause and CVD mortality, but with different characteristics.

**Registration:**

URL: https://clinicaltrials.gov/ct2/show/NCT00005275; Unique identifier: NCT00005275.

**Nonstandard Abbreviations and Acronyms**
ARIC: Atherosclerosis Risk in Communities
CHS: Cardiovascular Health Study
SHHS: Sleep Heart Health Study
SHS: Strong Heart Study
T_90_
: oxygen desaturation <90%

**Clinical Perspective**

**What Is New?**

- Self‐reported and objective sleep duration had different effects on the all‐cause and cardiovascular disease mortality.
- There is a J‐shaped association of self‐reported sleep duration on weekdays and weekends with all‐cause and cardiovascular disease mortality.
- Objective sleep duration is inversely associated with all‐cause and cardiovascular disease mortality.

**What Are the Clinical Implications?**

- Self‐reported sleep duration may not fully reflect people's sleep duration.
- Objective sleep monitoring is necessary and important to identify the real sleep duration and fluctuations.

Sleep is a complicated set of brain processes that plays a pivotal role in maintaining mental and physical health.^1^ Insufficient sleep can lead to multiple adverse medical consequences, including cardiovascular diseases (CVDs),^2^, ^3^ metabolic disorders,^4^ psychiatric disorders,^5^ and impaired cognition.^6^, ^7^ Sleep duration is an important indicator of nighttime sleep, which can be objectively measured using polysomnography and wrist actigraphy or collected from self‐reported sleep questionnaires.

Accumulating evidence indicates that sleep duration is strongly associated with mortality; however, most studies have focused on the effects of self‐reported sleep duration.^8^, ^9^ There is often a discrepancy between self‐reported and objective sleep duration assessments caused by sleep misperception.^10^ Self‐reported sleep duration generally overestimated objectively measured sleep in previous observational studies.^11^, ^12^ A U‐shaped or J‐shaped association has been observed between self‐reported sleep duration and all‐cause and CVD mortality.^13^, ^14^ Reinhard et al showed that objective sleep duration was inversely correlated with mortality in patients with chronic heart failure.^15^

To our knowledge, there is little evidence comparing the different characteristics of self‐reported and objective sleep duration on all‐cause and CVD mortality in the general population. The present study was designed to investigate the role of objective and self‐reported sleep duration in all‐cause and CVD mortality based on a community‐based population from the decade‐long SHHS (Sleep Heart Health Study).

## Methods

### Data Sharing Statement

The data used in this study were obtained from SHHS data sets (https://doi.org/10.25822/ghy8‐ks59).

### Study Population

The SHHS is a community‐based multicenter cohort study (participants enrolled between November 1, 1995, and January 31, 1998) with a primary aim of investigating the cardiovascular and other consequences of sleep‐disordered breathing (ClinicalTrials.gov Identifier: NCT00005275). A total of 6441 men and women 40 years and older were enrolled from large “parent” cohorts (including the Framingham Offspring Study; the Hagerstown and Minneapolis/St. Paul sites of the ARIC [Atherosclerosis Risk in Communities] study; the Hagerstown, Sacramento, and Pittsburgh sites of the CHS [Cardiovascular Health Study]; the SHS [Strong Heart Study] sites in South Dakota, Oklahoma, and Arizona; and studies of respiratory disease in Tucson and of hypertension in New York). Outcomes data such as all‐cause mortality, coronary artery disease and stroke were monitored for more than 10 years (between baseline and 2011). Details of the study design and quality control procedures have been previously reported.^16^, ^17^, ^18^ All of the participants in the SHHS provided written informed consent. The exclusion criteria were as follows: (1) 637 participants from the SHS were excluded because of sovereignty issues (SHS participants are not included in the shared SHHS data); and (2) 777 participants were excluded because of missing follow‐up data (the New York University‐Cornell site did not have outcome data). Ultimately, 5027 participants were included in the analysis.

### Objective and Self‐Reported Sleep Duration

All participants in the present study underwent electroencephalography‐based overnight unattended polysomnography (P‐Series, Compumedics) at their residence. The sleep monitor was set up by trained and certified technicians. Objective sleep duration, acquired from the polysomnography records, was defined as the total sleep time consisting of rapid eye movement sleep and non‐REM sleep. If the polysomnography monitoring was inadequate, a repeat monitoring request was initiated (Data S1).

Self‐reported sleep duration was obtained from sleep habit questionnaires such as “How many hours of sleep do you usually get at night (or What time do you usually fall asleep and wake up) on weekdays or workdays?” and “How many hours of sleep do you usually get at night (or What time do you usually fall asleep and wake up) on weekends or your nonworkdays?” We also collected the sleep duration from the morning survey based on the question “How long did you sleep last night?” Sleep duration was further categorized as ≤4 hours, 4 to 5 hours, 5 to 6 hours, 6 to 7 hours, 7 to 8 hours, and >8 hours in this study.

### Covariates

Baseline characteristics of the study sample, including age, sex, race, body weight, smoking status, alcohol use, medical history, and medication use, were acquired from the SHHS baseline survey and parent study. The apnea‐hypopnea index was defined as all episodes of apnea and hypopnea per hour associated with an oxygen desaturation of ≥4%. The percentage of sleep time with oxygen desaturation <90% (T_90_) was defined as the ratio of time spent with oxygen desaturation <90% of the total sleep time.

### Outcomes

Deaths from any cause were assessed in the parent study, which was identified and confirmed by follow‐up interviews. When a participant could not be reached for a scheduled follow‐up, all known contacts of the participant were called to determine the participant's survival status. All deaths including CVD death were investigated by reviewing local hospital records, including ECGs, heart catheterizations, cardiac surgery, echocardiography, nuclear medicine scans, radiographs, computed tomography and magnetic resonance imaging, cerebral angiograms, lumbar punctures, pathology reports, death certificates, autopsy reports, and laboratory tests. In addition, the participant's physician and the family members or other proxies who were with the participant at the time of death were interviewed to obtain detailed information about the circumstances of their death. Community obituaries were also monitored and linked to the Social Security Administration Death Master File.^19^

### Statistical Analysis

Comparisons of baseline characteristics are presented as mean±SD for continuous variables and number (percentages) for categorical variables. Differences in the characteristics were analyzed using chi‐square test for categorical variables and independent Student *t* test for continuous variables. Unadjusted Kaplan–Meier plots were used to evaluate the overall survival of different sleep duration categories. Multivariable Cox proportional hazard regression models were used to assess the association between objective and self‐reported sleep duration categories and all‐cause and CVD mortality. Covariates were selected in our multivariable analysis based on: (1) the variables considered clinically relevant or used in previous study; and (2) the variable was significantly associated with mortality in univariate analysis (*P*<0.05). The model was adjusted for age, sex, race, body mass index, smoking status, alcohol use, diabetes, hypertension, history of major CVD (including myocardial infarction, congestive heart failure, and stroke), history of chronic respiratory disease (including chronic obstructive pulmonary disease and chronic bronchitis), lipid‐lowering medication use, benzodiazepine use, apnea‐hypopnea index, and T_90_.

Restricted cubic spline Cox regression was applied to assess the dose–response association of objective and self‐reported sleep duration with all‐cause and CVD mortality (5 knots at the 5.0th, 27.5th, 50.0th, 72.5th, and 95.0th percentiles). Pearson correlation was used to determine the correlation between objective and self‐reported sleep duration. All statistical analyses were performed using SPSS statistics software (version 24.0, IBM) and R software version 3.6.3 (R Core Team). All statistical significance was 2‐sided, and a *P* value <0.05 was considered statistically significant.

## Results

### Baseline Characteristics of Participants

A total of 5027 participants (2341 men and 2686 women [mean age, 63.9±11.1 years]) were enrolled in the present study. Compared with people alive, individuals with all‐cause death had shorter objective sleep duration (5.7±1.1 hours versus 6.1±1.0 hours, *P*<0.001) and longer self‐reported sleep duration on weekdays (7.6±1.4 hours versus 7.3±1.1 hours, *P*<0.001) (Table 1). No significant difference was observed between the number of people alive and all‐cause deaths in self‐reported sleep duration on weekends. Additionally, objective sleep duration weakly correlated with self‐reported sleep duration (Figure S1).

**Table 1:** Baseline Characteristics of the Study Sample According to Alive and All‐Cause Death

Characteristics	Total (N=5027)	All‐cause death (n=1172)	Alive (n=3855)	*P* value
Age, y	63.9±11.1	73.5±8.9	61.0±10.1	<0.001
Sex, n (%)				<0.001
Men	2341 (46.6)	622 (35.1)	1719 (44.6)	—
Women	2686 (53.4)	550 (46.9)	2136 (55.4)	—
Race and ethnicity, n (%)*				0.320
White	4375 (87.0)	1030 (87.9)	3345 (86.8)	—
Other	652 (13.0)	142 (12.1)	510 (13.2)	—
Body weight, n (%)				<0.001
Obesity	1540 (30.9)	315 (27.2)	1225 (32.0)	—
Overweight	2121 (42.6)	488 (42.2)	1633 (42.7)	—
Normal	1320 (26.5)	353 (30.6)	967 (25.3)	—
Smoking status, n (%)				<0.001
Current	487 (9.7)	114 (9.7)	373 (9.7)	—
Former	2210 (44.1)	576 (49.3)	1634 (42.5)	—
Never	2316 (46.2)	479 (41.0)	1837 (47.8)	—
Alcohol use, n (%)				<0.001
At least 1 drink per day	2014 (42.8)	404 (35.5)	1610 (45.1)	—
None	2691 (57.2)	733 (64.5)	1958 (54.9)	—
Medical history, n (%)				
MI	341 (6.8)	177 (15.1)	164 (4.3)	<0.001
CHF	140 (2.8)	101 (8.6)	39 (1.0)	<0.001
Stroke	153 (3.0)	70 (6.0)	83 (2.2)	<0.001
COPD	59 (1.2)	27 (2.3)	32 (0.8)	<0.001
Chronic bronchitis	75 (1.5)	92 (7.8)	7 (0.2)	<0.001
Diabetes	367 (7.3)	179 (15.3)	188 (4.9)	<0.001
Hypertension	2031 (40.4)	713 (60.8)	1318 (34.2)	<0.001
Lipid‐lowering medication use, n (%)	634 (12.6)	165 (14.1)	469 (12.2)	0.084
Benzodiazepine use, n (%)	284 (5.6)	94 (8.0)	190 (4.9)	<0.001
Sleep duration, h				
Objective	6.0±1.1	5.7±1.1	6.1±1.0	<0.001
Self‐reported weekday	7.4±1.2	7.6±1.4	7.3±1.1	<0.001
Self‐reported weekend	7.7±1.3	7.7±1.5	7.7±1.2	0.871
AHI, events/h	10.1±13.4	12.4±15.0	9.4±12.8	<0.001
T_90_, %	3.6±10.6	6.4±15.2	2.8±8.6	<0.001
Follow‐up time, y	10.7±3.0	6.9±3.2	11.9±1.7	<0.001

Results are presented as mean±SD or number (percentage). The *P* values represent the difference between the 2 groups. AHI indicates apnea‐hypopnea index; CHF, congestive heart failure; COPD, chronic obstructive pulmonary disease; MI, myocardial infarction; and T_90_, oxygen desaturation <90%.

* White did not include Hispanic or Latino participants. Other race and ethnicity categories included Black, Native American or Alaskan Native, Asian or Pacific Islander, and Hispanic or Latino.

### Association of Objective Sleep Duration With All‐Cause and CVD Mortality

**Figure 1:**
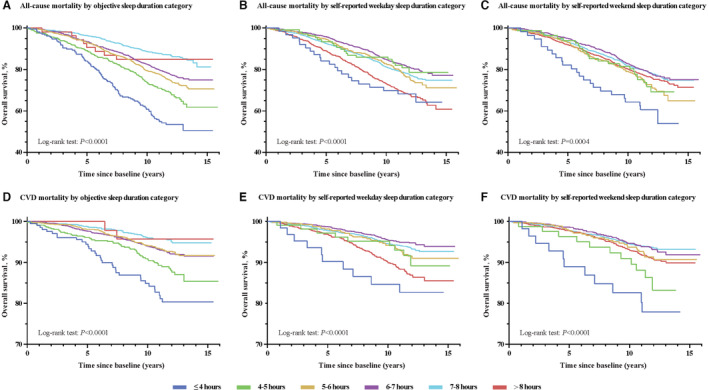
Kaplan–Meier plots of overall survival for all‐cause and cardiovascular disease (CVD) mortality by objective and self‐reported sleep duration. **A**, Objective sleep duration category and all‐cause mortality. **B**, Self‐reported sleep duration category on weekdays and all‐cause mortality. **C**, Self‐reported sleep duration category on weekends and all‐cause mortality. **D**, Objective sleep duration category and CVD mortality. **E**, Self‐reported sleep duration category on weekdays and CVD mortality. **F**, Self‐reported sleep duration category on weekends and CVD mortality.

During an average of 10.7±3.0 years of follow‐up, 1172 (23.3%) all‐cause deaths were identified, including 359 (7.1%) CVD deaths. Individuals with objective sleep duration of ≤4 hours (45.9%), 4 to 5 hours (31.3%), 5 to 6 hours (25.0%), and 6 to 7 hours (21.9%) had a higher all‐cause mortality than those with a sleep duration of 7 to 8 hours (13.5%). All‐cause mortality was similar for the objective sleep duration at 7 to 8 hours and >8 hours. Unadjusted Kaplan–Meier analysis showed lower overall survival among participants with a shorter objective sleep duration (Figure 1).

**Figure 2:**
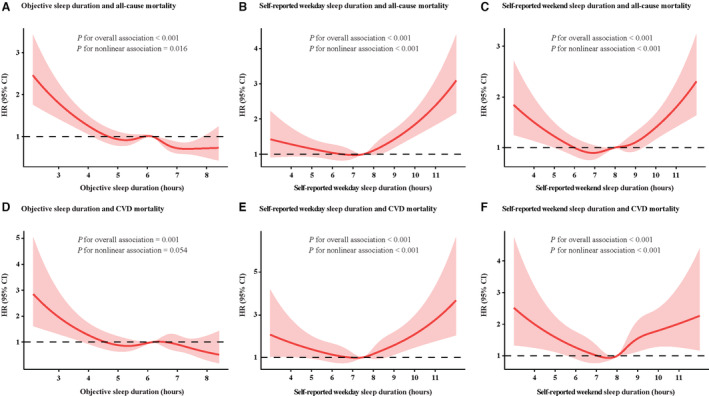
Multivariable Cox proportional hazard ratio (HR) for all‐cause and cardiovascular disease (CVD) mortality based on restricted cubic spline analysis of sleep duration. **A**, Objective sleep duration and all‐cause mortality. **B**, Self‐reported sleep duration on weekdays and all‐cause mortality. **C**, Self‐reported sleep duration on weekends and all‐cause mortality. **D**, Objective sleep duration and CVD mortality. **E**, Self‐reported sleep duration on weekdays and CVD mortality. **F**, Self‐reported sleep duration on weekends and CVD mortality.

After adjusting for age, sex, race, body mass index, smoking status, alcohol use, diabetes, hypertension, history of major CVD, history of chronic respiratory disease, lipid‐lowering medication use, benzodiazepine use, apnea‐hypopnea index, and T_90_, multivariable Cox regression analysis found that objective sleep duration ≤4 hours (hazard ratio [HR], 2.43 [95% CI, 1.83–3.22], *P*<0.001), 4 to 5 hours (HR, 1.66 [95% CI, 1.30–2.12], *P*<0.001), 5 to 6 hours (HR, 1.37 [95% CI, 1.10–1.71], *P*=0.005), and 6 to 7 hours (HR, 1.42 [95% CI, 1.15–1.76], *P*=0.001) were significantly associated with all‐cause mortality compared with that of the reference (7–8 hours) (Table 2). In the restricted cubic spline analysis, an increasing objective sleep duration was accompanied by an overall downward trend in all‐cause mortality (*P*_overall association_<0.001; *P*_nonlinear association_=0.016; Figure 2).

**Table 2:** HRs and 95% CIs for Objective and Self‐Reported Sleep Duration Associated With All‐Cause Mortality

					Univariate model		Multivariable adjusted†		Multivariable adjusted‡	
Sleep duration	Patients, n	Events, n (%)	Person‐years	Mortality*	HR (95% CI)	*P* value	HR (95% CI)	*P* value	HR (95% CI)	*P* value
Objective sleep duration, h										
≤4	207	95 (45.9)	1934.2	49.1	4.29 (3.27–5.63)	<0.001	2.82 (2.15–3.71)	<0.001	2.43 (1.83–3.22)	<0.001
4–5	582	182 (31.3)	5876.4	31.0	2.65 (2.10–3.35)	<0.001	1.76 (1.39–2.23)	<0.001	1.66 (1.30–2.12)	<0.001
5–6	1396	349 (25.0)	14,905.9	23.4	1.92 (1.60–2.45)	<0.001	1.48 (1.19–1.83)	<0.001	1.37 (1.10–1.71)	0.005
6–7	1933	423 (21.9)	20,848.7	20.3	1.71 (1.39–2.10)	<0.001	1.43 (1.17–1.77)	0.001	1.42 (1.15–1.76)	0.001
7–8	855	115 (13.5)	9617.4	12.0	1 (Ref)		1 (Ref)		1 (Ref)	
>8	54	8 (14.8)	599.1	13.4	1.12 (0.55–2.30)	0.749	1.70 (0.83–3.48)	0.148	1.63 (0.79–3.34)	0.187
Self‐reported weekday sleep duration, h										
≤4	63	21 (33.3)	630.2	33.3	1.71 (1.10–2.66)	0.017	1.74 (1.12–2.70)	0.014	1.63 (1.04–2.54)	0.032
4–5	108	21 (19.4)	1164.8	18.0	0.91 (0.59–1.42)	0.689	0.85 (0.55–1.31)	0.454	0.68 (0.43–1.08)	0.101
5–6	513	119 (23.2)	5465.3	21.8	1.11 (0.90–1.36)	0.322	1.08 (0.88–1.33)	0.471	1.02 (0.82–1.26)	0.866
6–7	1465	275 (18.8)	16,180.6	17.0	0.86 (0.74–1.00)	0.053	0.91 (0.78–1.06)	0.229	0.93 (0.79–1.09)	0.340
7–8	1791	384 (21.4)	19,437.9	19.7	1 (Ref)		1 (Ref)		1 (Ref)	
>8	986	313 (31.7)	9950.7	31.5	1.62 (1.40–1.88)	<0.001	1.37 (1.18–1.59)	<0.001	1.26 (1.08–1.47)	0.003
Self‐reported weekend sleep duration, h										
≤4	57	23 (40.4)	534.6	43.0	2.21 (1.45–3.37)	<0.001	1.90 (1.25–2.90)	0.003	1.89 (1.23–2.89)	0.004
4–5	83	23 (27.7)	870.1	26.4	1.33 (0.87–2.02)	0.191	1.09 (0.72–1.67)	0.681	0.96 (0.62–1.50)	0.857
5–6	373	103 (27.6)	3916.2	26.3	1.32 (1.06–1.65)	0.012	1.17 (0.94–1.45)	0.169	1.10 (0.88–1.38)	0.421
6–7	1108	238 (21.5)	12,084.7	19.7	0.98 (0.83–1.16)	0.827	0.94 (0.79–1.10)	0.419	0.94 (0.80–1.12)	0.501
7–8	1704	369 (21.7)	18,390.2	20.1	1 (Ref)		1 (Ref)		1 (Ref)	
>8	1602	380 (23.7)	17,030.4	22.3	1.12 (0.97–1.29)	0.131	1.21 (1.05–1.39)	0.010	1.16 (1.01–1.35)	0.046

HR indicates hazard ratio.

* Crude event rate per 1000 person‐years.

^†^ Adjusted by age and sex.

^‡^ Adjusted by age, race, body mass index, smoking status, alcohol use, diabetes, hypertension, history of major cardiovascular disease (myocardial infarction, congestive heart failure, and stroke), history of chronic respiratory disease (chronic obstructive pulmonary disease, chronic bronchitis), lipid‐lowering medication use, benzodiazepine use, apnea‐hypopnea index, and oxygen desaturation <90%.

In addition, individuals with a short objective sleep duration had high CVD mortality rates (Figure 1). Multivariable Cox regression analysis showed that a short objective sleep duration of ≤4 hours (HR, 2.31 [95% CI, 1.43–3.75], *P*=0.001) and 4 to 5 hours (HR, 1.57 [95% CI, 1.03–2.40], *P*=0.038) had a high rate of CVD mortality compared with those of 7 to 8 hours (Table 3). Moreover, an inverse linear association between objective sleep duration and CVD death was observed in the restricted cubic spline analysis (*P*_overall association_<0.001; *P*_nonlinear association_=0.054; Figure 2).

**Table 3:** HRs and 95% CIs for Objective and Self‐Reported Sleep Duration Associated With CVD Mortality

					Univariate model		Multivariable adjusted†		Multivariable adjusted‡	
Sleep duration	Patients, n	Events, n (%)	Person‐years	Mortality*	HR (95% CI)	*P* value	HR (95% CI)	*P* value	HR (95% CI)	*P* value
Objective sleep duration, h										
≤4	207	33 (15.9)	1934.2	17.1	4.49 (2.82–7.16)	<0.001	2.78 (1.74–4.44)	<0.001	2.31 (1.43–3.75)	0.001
4–5	582	60 (10.3)	5876.4	10.2	2.63 (1.75–3.95)	<0.001	1.63 (1.08–2.46)	0.019	1.57 (1.03–2.40)	0.038
5–6	1396	92 (6.6)	14,905.9	6.2	1.58 (1.08–2.30)	0.018	1.11 (0.76–1.63)	0.596	1.06 (0.72–1.58)	0.757
6–7	1933	134 (6.9)	20,848.7	6.4	1.63 (1.14–2.34)	0.008	1.32 (0.92–1.90)	0.132	1.35 (0.93–1.96)	0.112
7–8	855	38 (4.4)	9617.4	4.0	1 (Ref)		1 (Ref)		1 (Ref)	
>8	54	2 (3.7)	599.1	3.3	0.86 (0.21–3.54)	0.829	1.39 (0.34–5.76)	0.651	1.25 (0.30–5.22)	0.760
Self‐reported weekday sleep duration, h										
≤ 4	63	10 (15.9)	630.2	15.9	2.91 (1.52–5.56)	0.001	2.98 (1.56–5.70)	0.001	2.56 (1.32–4.95)	0.005
4–5	108	9 (8.3)	1164.8	7.7	1.40 (0.71–2.77)	0.329	1.28 (0.65–2.54)	0.471	0.94 (0.45–1.94)	0.865
5–6	513	37 (7.2)	5465.3	6.8	1.24 (0.85–1.80)	0.260	1.20 (0.82–1.74)	0.345	1.07 (0.72–1.58)	0.749
6–7	1465	72 (4.9)	16,180.6	4.4	0.81 (0.60–1.09)	0.159	0.86 (0.64–1.16)	0.316	0.95 (0.70–1.29)	0.749
7–8	1791	107 (6.0)	19,437.9	5.5	1 (Ref)		1 (Ref)		1 (Ref)	
>8	986	108 (11.0)	9950.7	10.9	2.01 (1.54–2.62)	<0.001	1.66 (1.27–2.17)	<0.001	1.53 (1.16–2.02)	0.002
Self‐reported weekend sleep duration, h										
≤4	57	11 (19.3)	534.6	20.6	3.92 (2.10–7.31)	<0.001	3.33 (1.79–6.22)	<0.001	3.12 (1.66–5.87)	<0.001
4–5	83	11 (13.3)	870.1	12.6	2.35 (1.26–4.38)	0.007	1.91 (1.03–3.57)	0.042	1.41 (0.71–2.81)	0.330
5–6	373	28 (7.5)	3916.2	7.1	1.34 (0.88–2.04)	0.174	1.16 (0.77–1.77)	0.480	1.08 (0.69–1.68)	0.743
6–7	1108	68 (6.1)	12,084.7	5.6	1.05 (0.77–1.43)	0.772	0.99 (0.73–1.35)	0.954	1.11 (0.81–1.52)	0.530
7–8	1704	99 (5.8)	18,390.2	5.4	1 (Ref)		1 (Ref)		1 (Ref)	
>8	1602	128 (8.0)	17,030.4	7.5	1.41 (1.08–1.83)	0.011	1.53 (1.18–1.99)	0.001	1.55 (1.18–2.03)	0.001

HR indicates hazard ratio.

* Crude event rate per 1000 person‐years.

^†^ Adjusted by age and sex.

^‡^ Adjusted by age, race, body mass index, smoking status, alcohol use, diabetes, hypertension, history of major cardiovascular disease (myocardial infarction, congestive heart failure, and stroke), history of chronic respiratory disease (chronic obstructive pulmonary disease, chronic bronchitis), lipid‐lowering medication use, benzodiazepine use, apnea‐hypopnea index, and oxygen desaturation <90%.

### Association of Self‐Reported Sleep Duration With All‐Cause and CVD Mortality

Unlike objective sleep, both short (≤4 hours) and long (>8 hours) self‐reported sleep duration on weekdays had a high rate of all‐cause mortality (Table 2; Figure 1). Compared with a self‐reported weekday sleep duration of 7 to 8 hours, weekday sleep duration of ≤4 hours (HR, 1.63 [95% CI, 1.04–2.54], *P*=0.032) and >8 hours (HR, 1.26 [95% CI, 1.08–1.47], *P*=0.003) were associated with an increased risk of all‐cause mortality. A J‐shaped association was found between self‐reported sleep duration on weekdays and all‐cause mortality (*P*_overall association_<0.001; *P*_nonlinear association_<0.001; Figure 2).

The association of short (≤4 hours) and long (>8 hours) self‐reported sleep duration on weekdays with a high CVD mortality rate was also observed. We also found a J‐shaped curve between self‐reported sleep duration on weekdays and all‐cause mortality (*P*_overall association_<0.001; *P*_nonlinear association_<0.001; Figure 2). Moreover, self‐reported sleep duration on weekends had a similar effect on all‐cause and CVD mortality as that of weekday sleep duration.

The association of objective and self‐reported sleep duration with all‐cause and CVD mortality in men and women was also explored. There was an inverse association between the objective sleep duration and mortality in both men and women. A J‐shaped association was found between self‐reported sleep duration and mortality in both men and women (Figures S2 and S3). We further investigated the association between sleep duration based on morning survey and mortality. However, no significant association was found (Table S1).

## Discussion

In this large community‐based study, we investigated the role of objective and self‐reported sleep duration in all‐cause and CVD mortality. The effects of objective and self‐reported sleep duration on mortality differed. A J‐shaped association of self‐reported sleep duration on weekdays and weekends with all‐cause and CVD mortality was observed. We also found that all‐cause and CVD mortality rates decreased gradually with increasing objective sleep duration.

Research on the relationship between sleep duration and all‐cause and CVD mortality is inconsistent.^20^, ^21^, ^22^ A growing number of studies have demonstrated a U‐shaped or J‐shaped association of self‐reported sleep duration with all‐cause and CVD mortality.^13^, ^22^, ^23^ Both short and long self‐reported sleep duration were associated with a high risk of all‐cause and CVD mortality.^24^, ^25^, ^26^, ^27^ Moreover, a U‐shaped association between self‐reported sleep duration on weekdays and weekends and all‐cause mortality was observed in a large population study.^28^ In our study, we explored the association between self‐reported sleep duration on both weekdays and weekends and all‐cause and CVD mortality. A J‐shaped association was observed between self‐reported sleep duration on weekdays and weekends and all‐cause and CVD mortality. Our findings indicate that self‐reported weekday and weekend sleep duration of 7 to 8 hours were the nadir for associations with all‐cause and CVD mortality.

Sleep questionnaires, commonly used to evaluate self‐reported sleep duration in epidemiological studies, do not correspond closely to objectively measured sleep duration.^29^, ^30^ Several studies have demonstrated that self‐reported sleep duration tends to overestimate objectively measured sleep duration, which may be attributable to sleep misperceptions.^10^, ^11^ We also found a higher self‐reported sleep duration than objective sleep duration. Moreover, our findings show a weak correlation between objective and self‐reported sleep duration. In the present study, we sought to observe the different effects of self‐reported sleep duration and objective sleep duration on all‐cause and CVD mortality. One observational study with a small sample size showed an inverse association between objective sleep duration and all‐cause mortality in patients with chronic heart failure.^15^ Bertisch et al demonstrated that insomnia or poor sleep with objective short sleep duration (<6 hours) was associated with higher risk of incident CVD but not with all‐cause mortality.^31^ We utilized restricted cubic spline analysis to investigate the dose–response association of objective sleep duration with all‐cause and CVD mortality in a community‐based population. Our findings reveal that the association between objective sleep duration and mortality was completely different from that of self‐reported sleep duration. With an increase in the objective sleep duration, the rates of all‐cause and CVD mortality showed a significant downward trend. Interestingly, the rates of all‐cause and CVD mortality were lower in both objective and self‐reported sleep duration of 7 to 8 hours.

The biological mechanisms underlying the potential association between sleep duration and all‐cause and CVD mortality are not yet fully understood. Our findings reveal that objectively measured and self‐reported short sleep duration had an obviously high rate of all‐cause and CVD mortality. It may be because short sleep duration has been linked to 7 (including CVD, malignant neoplasm, cerebrovascular disease, accidents, diabetes, septicemia, and hypertension) of the 15 leading causes of death in America.^32^ Moreover, short sleep duration is related to reduced leptin and elevated ghrelin levels.^33^ Perturbation of leptin and ghrelin levels may be related to increased appetite and calorie intake, which could lead to the development of obesity and diabetes, ultimately increasing mortality. Furthermore, increased all‐cause and CVD mortality was observed only in the long self‐reported sleep duration group. This may be attributable to the fact that individuals with long self‐reported sleep duration had significantly increased sleep latency, wake after sleep onset, and arousal index (Table S2). These findings remind us that self‐reported long sleep duration may include more wakefulness time in bed. In addition, wake after sleep onset was also found to be associated with both all‐cause and CVD mortality (Table S3). Increased wake after sleep onset may be the underlying cause of higher mortality in people who had self‐reported long sleep duration. Moreover, long sleep duration may be related to fatigue, immune function, photoperiodic abnormalities, depression, and some underlying diseases.^34^

The current study, based on a community‐based population, reported the association of objective and self‐reported sleep duration with all‐cause and CVD mortality that may be available to the general population. We highlight the different characteristics of the effects of objective and self‐reported sleep duration on mortality. This study had several limitations. Most of the individuals were of White race and middle‐aged or older; our findings may not apply to other ethnic groups or younger individuals. We did not include socioeconomic status because of a lack of relevant information in SHHS data sets. Moreover, the objective sleep duration in this study was monitored using single‐night in‐home polysomnography. Nocturnal variability in sleep duration may exist and affect the strength of observed associations.

## Conclusions

The current study indicates that the effects of objective and self‐reported sleep duration on mortality are different in middle‐aged and older people. Objective sleep duration tended to have an inverse association with all‐cause and CVD mortality. A J‐shaped association was observed between self‐reported sleep duration and mortality. A short sleep duration in both objectively measured and self‐reported data was associated with increased all‐cause and CVD mortality. Insufficient sleep is an important public health concern. Self‐reported long sleep duration is also a concern, especially if it is caused by sleep fragmentation and underlying diseases. Importantly, self‐reported sleep duration is the most practical and cost‐effective research method, but it may not fully reflect real sleep duration. Long‐term monitoring of objective sleep duration can reflect the true level and fluctuation of sleep duration, and the development and popularization of smart wearable devices may provide a convenient means of collecting these data for studies in the near future.

## Sources of Funding

This work was supported by the Natural Science Basic Research Program of Shaanxi (No. 2021JQ‐395).

## Disclosures

All of the authors declare no conflicts of interest.

## Supporting information

Data S1
Tables S1–S3
Figures S1–S3

## References

[1] Kirszenblat L , van Swinderen B . The yin and yang of sleep and attention. Trends Neurosci. 2015;38:776–786. doi: 10.1016/j.tins.2015.10.001 26602764 PMC4803435

[2] Khan H , Kella D , Kunutsor SK , Savonen K , Laukkanen JA . Sleep duration and risk of fatal coronary heart disease, sudden cardiac death, cancer death, and all‐cause mortality. Am J Med. 2018;131:1499–1505.e2. doi: 10.1016/j.amjmed.2018.07.010 30076817

[3] Fan M , Sun D , Zhou T , Heianza Y , Lv J , Li L , Qi L . Sleep patterns, genetic susceptibility, and incident cardiovascular disease: a prospective study of 385 292 UK biobank participants. Eur Heart J. 2020;41:1182–1189. doi: 10.1093/eurheartj/ehz849 31848595 PMC7071844

[4] Hall MH , Muldoon MF , Jennings JR , Buysse DJ , Flory JD , Manuck SB . Self‐reported sleep duration is associated with the metabolic syndrome in midlife adults. Sleep. 2008;31:635–643. doi: 10.1093/sleep/31.5.635 18517034 PMC2398755

[5] Zhang MM , Ma Y , Du LT , Wang K , Li Z , Zhu W , Sun YH , Lu L , Bao YP , Li SX . Sleep disorders and non‐sleep circadian disorders predict depression: a systematic review and meta‐analysis of longitudinal studies. Neurosci Biobehav Rev. 2022;134:104532. doi: 10.1016/j.neubiorev.2022.104532 35041878

[6] Winer JR , Deters KD , Kennedy G , Jin M , Goldstein‐Piekarski A , Poston KL , Mormino EC . Association of short and long sleep duration with amyloid‐beta burden and cognition in aging. JAMA Neurol. 2021;78:1187–1196. doi: 10.1001/jamaneurol.2021.2876 34459862 PMC8406215

[7] Lucey BP , Wisch J , Boerwinkle AH , Landsness EC , Toedebusch CD , McLeland JS , Butt OH , Hassenstab J , Morris JC , Ances BM , et al. Sleep and longitudinal cognitive performance in preclinical and early symptomatic Alzheimer's disease. Brain. 2021;144:2852–2862. doi: 10.1093/brain/awab272 34668959 PMC8536939

[8] Yin J , Jin X , Shan Z , Li S , Huang H , Li P , Peng X , Peng Z , Yu K , Bao W , et al. Relationship of sleep duration with all‐cause mortality and cardiovascular events: a systematic review and dose‐response meta‐analysis of prospective cohort studies. J Am Heart Assoc. 2017;6:6. doi: 10.1161/JAHA.117.005947 PMC5634263 28889101

[9] da Silva AA , de Mello RG , Schaan CW , Fuchs FD , Redline S , Fuchs SC . Sleep duration and mortality in the elderly: a systematic review with meta‐analysis. BMJ Open. 2016;6:e008119. doi: 10.1136/bmjopen-2015-008119 PMC4762152 26888725

[10] Silva GE , Goodwin JL , Sherrill DL , Arnold JL , Bootzin RR , Smith T , Walsleben JA , Baldwin CM , Quan SF . Relationship between reported and measured sleep times: the sleep heart health study (SHHS). J Clin Sleep Med. 2007;3:622–630. doi: 10.5664/jcsm.26974 17993045 PMC2045712

[11] Jackson CL , Patel SR , Jackson WB II , Lutsey PL , Redline S . Agreement between self‐reported and objectively measured sleep duration among white, black, Hispanic, and Chinese adults in the United States: Multi‐Ethnic Study of Atherosclerosis. Sleep. 2018;41:zsy057. doi: 10.1093/sleep/zsy057 29701831 PMC5995218

[12] Lauderdale DS , Knutson KL , Yan LL , Liu K , Rathouz PJ . Self‐reported and measured sleep duration: how similar are they? Epidemiology. 2008;19:838–845. doi: 10.1097/EDE.0b013e318187a7b0 18854708 PMC2785092

[13] Wang C , Bangdiwala SI , Rangarajan S , Lear SA , AlHabib KF , Mohan V , Teo K , Poirier P , Tse LA , Liu Z , et al. Association of estimated sleep duration and naps with mortality and cardiovascular events: a study of 116 632 people from 21 countries. Eur Heart J. 2019;40:1620–1629. doi: 10.1093/eurheartj/ehy695 30517670 PMC6528160

[14] Svensson T , Saito E , Svensson AK , Melander O , Orho‐Melander M , Mimura M , Rahman S , Sawada N , Koh WP , Shu XO , et al. Association of sleep duration with all‐ and major‐cause mortality among adults in Japan, China, Singapore, and Korea. JAMA Netw Open. 2021;4:e2122837. doi: 10.1001/jamanetworkopen.2021.22837 34477853 PMC8417759

[15] Reinhard W , Plappert N , Zeman F , Hengstenberg C , Riegger G , Novack V , Maimon N , Pfeifer M , Arzt M . Prognostic impact of sleep duration and sleep efficiency on mortality in patients with chronic heart failure. Sleep Med. 2013;14:502–509. doi: 10.1016/j.sleep.2012.12.014 23628241

[16] Quan SF , Howard BV , Iber C , Kiley JP , Nieto FJ , O'Connor GT , Rapoport DM , Redline S , Robbins J , Samet JM , et al. The Sleep Heart Health Study: design, rationale, and methods. Sleep. 1997;20:1077–1085. 9493915

[17] Redline S , Sanders MH , Lind BK , Quan SF , Iber C , Gottlieb DJ , Bonekat WH , Rapoport DM , Smith PL , Kiley JP . Methods for obtaining and analyzing unattended polysomnography data for a multicenter study. Sleep Heart Health Research Group. Sleep. 1998;21:759–767. 11300121

[18] Zhang GQ , Cui L , Mueller R , Tao S , Kim M , Rueschman M , Mariani S , Mobley D , Redline S . The National Sleep Research Resource: towards a sleep data commons. J Am Med Inform Assoc. 2018;25:1351–1358. doi: 10.1093/jamia/ocy064 29860441 PMC6188513

[19] Punjabi NM , Caffo BS , Goodwin JL , Gottlieb DJ , Newman AB , O'Connor GT , Rapoport DM , Redline S , Resnick HE , Robbins JA , et al. Sleep‐disordered breathing and mortality: a prospective cohort study. PLoS Med. 2009;6:e1000132. doi: 10.1371/journal.pmed.1000132 19688045 PMC2722083

[20] Kurina LM , McClintock MK , Chen JH , Waite LJ , Thisted RA , Lauderdale DS . Sleep duration and all‐cause mortality: a critical review of measurement and associations. Ann Epidemiol. 2013;23:361–370. doi: 10.1016/j.annepidem.2013.03.015 23622956 PMC3660511

[21] Grandner MA , Patel NP , Gehrman PR , Perlis ML , Pack AI . Problems associated with short sleep: bridging the gap between laboratory and epidemiological studies. Sleep Med Rev. 2010;14:239–247. doi: 10.1016/j.smrv.2009.08.001 19896872 PMC2888649

[22] Liu TZ , Xu C , Rota M , Cai H , Zhang C , Shi MJ , Yuan RX , Weng H , Meng XY , Kwong JS , et al. Sleep duration and risk of all‐cause mortality: a flexible, non‐linear, meta‐regression of 40 prospective cohort studies. Sleep Med Rev. 2017;32:28–36. doi: 10.1016/j.smrv.2016.02.005 27067616

[23] Guo QY , Xie WH , Peng R , Ma Y , Chong FF , Wang YL , Song MM , Ye H , Wang P , Wang KJ , et al. A dose‐response relationship between sleep duration and stroke according to nonhealth status in Central China: a population‐based epidemiology survey. J Stroke Cerebrovasc. 2019;28:1841–1852. doi: 10.1016/j.jstrokecerebrovasdis.2019.04.016 31076320

[24] Yang LL , Xi B , Zhao M , Magnussen CG . Association of sleep duration with all‐cause and disease‐specific mortality in US adults. J Epidemiol Commun Health. 2021;75:556–561. doi: 10.1136/jech-2020-215314 33441393

[25] Kronholm E , Laatikainen T , Peltonen M , Sippola R , Partonen T . Self‐reported sleep duration, all‐cause mortality, cardiovascular mortality and morbidity in Finland. Sleep Med. 2011;12:215–221. doi: 10.1016/j.sleep.2010.07.021 21317033

[26] Tao F , Cao Z , Jiang Y , Fan N , Xu F , Yang H , Li S , Zhang Y , Zhang X , Sun L , et al. Associations of sleep duration and quality with incident cardiovascular disease, cancer, and mortality: a prospective cohort study of 407,500 UK biobank participants. Sleep Med. 2021;81:401–409. doi: 10.1016/j.sleep.2021.03.015 33819843

[27] Cappuccio FP , D'Elia L , Strazzullo P , Miller MA . Sleep duration and all‐cause mortality: a systematic review and meta‐analysis of prospective studies. Sleep. 2010;33:585–592. doi: 10.1093/sleep/33.5.585 20469800 PMC2864873

[28] Xiao Q , Blot WJ , Matthews CE . Weekday and weekend sleep duration and mortality among middle‐to‐older aged White and Black adults in a low‐income southern US cohort. Sleep Health. 2019;5:521–527. doi: 10.1016/j.sleh.2019.04.008 31204307 PMC6801047

[29] O'Brien E , Hart C , Wing RR . Discrepancies between self‐reported usual sleep duration and objective measures of total sleep time in treatment‐seeking overweight and obese individuals. Behav Sleep Med. 2016;14:539–549. doi: 10.1080/15402002.2015.1048447 26503348 PMC4848236

[30] Miner B , Stone KL , Zeitzer JM , Han L , Doyle M , Blackwell T , Gill TM , Redeker NS , Hajduk A , Yaggi HK . Self‐reported and actigraphic short sleep duration in older adults. J Clin Sleep Med. 2021;18:403–413. doi: 10.5664/jcsm.9584 PMC8804982 34338629

[31] Bertisch SM , Pollock BD , Mittleman MA , Buysse DJ , Bazzano LA , Gottlieb DJ , Redline S . Insomnia with objective short sleep duration and risk of incident cardiovascular disease and all‐cause mortality: Sleep Heart Health Study. Sleep. 2018;41:zsy047. doi: 10.1093/sleep/zsy047 29522193 PMC5995202

[32] Grandner MA , Hale L , Moore M , Patel NP . Mortality associated with short sleep duration: the evidence, the possible mechanisms, and the future. Sleep Med Rev. 2010;14:191–203. doi: 10.1016/j.smrv.2009.07.006 19932976 PMC2856739

[33] Taheri S , Lin L , Austin D , Young T , Mignot E . Short sleep duration is associated with reduced leptin, elevated ghrelin, and increased body mass index. Plos Med. 2004;1:210–217. doi: 10.1371/journal.pmed.0010062 PMC535701 15602591

[34] Grandner MA , Drummond SP . Who are the long sleepers? Towards an understanding of the mortality relationship. Sleep Med Rev. 2007;11:341–360. doi: 10.1016/j.smrv.2007.03.010 17625932 PMC3755488

