# Supplementary material for: Association of Objective and Self‐Reported Sleep Duration With All‐Cause and Cardiovascular Disease Mortality: A Community‐Based Study
Source: J Am Heart Assoc. 2023 Mar 9;12(6):e027832. doi: 10.1161/JAHA.122.027832 (PMC10111539; doi:10.1161/JAHA.122.027832)
Supplement: Supplementary file 1 — Data S1 Tables S1–S3 Figures S1–S3 [file JAH3-12-e027832-s001.pdf]

SUPPLEMENTAL MATERIAL

Data S1. Supplemental Methods

All the participants in the Sleep Heart Health Study (SHHS) underwent in-home polysomnography (PSG) to collect data of sleep parameters. Objective sleep duration was obtained from PSG records. The Compumedics sleep monitor was set up by a team of two individuals (one sleep technician and one helper) who have been specifically trained and certified. The sleep monitor is battery operated so the participant is not potentially in connection with electrical outlets. PSG records were obtained in an unattended setting. The next morning a technician returned to the participant's home at a pre-arranged time to collect the sleep monitor and self-administered surveys. The PSG data were then sent to the Reading Center for processing and the paper forms submitted for local data entry. The trained technicians completed manual analysis of monitoring results to obtain accurate information. If the study was inadequate, a repeat study request is initiated. The entire monitoring process tried to make participants consistent with their usual sleep. The each stage of sleep was clearly defined which could be found at https://sleepdata.org/datasets/shhs/pages/mop/6-626-mop-rules-for-assigning-sleep-stages.md.

A total of 6441 men and women aged 40 years or older completed the baseline examination (1995–1998). Institutional Review Board approval was obtained at all participating institutions, and all participants signed informed consent. Of the 6441 participants enrolled in SHHS, 637 withdrew their consent due to sovereignty issues

(Strong Heart Study participants are not included in the shared SHHS data). Therefore, SHHS dataset only includes the remaining 5804 participants. We also acquired 5804 participants from the SHHS datasets. The data could be found at https://doi.org/10.25822/ghy8-ks59.

Table S1. HRs and 95% CIs for self-reported sleep duration in morning survey associated with all-cause and CVD mortality

	**All-cause mortality**		**CVD mortality**	
Sleep duration, hours*	HR (95% CI)	*P*	HR (95% CI)	*P*
≤4	1.26 (0.97-1.62)	0.080	1.14 (0.71-1.82)	0.586
4-5	0.87 (0.69-1.11)	0.259	0.86 (0.57-1.32)	0.497
5-6	1.08 (0.91-1.30)	0.381	1.12 (0.81-1.54)	0.494
6-7	0.89 (0.75-1.06)	0.179	0.87 (0.64-1.18)	0.359
7-8	1 (Ref)		1 (Ref)	
>8	1.02 (0.83-1.25)	0.849	0.91 (0.63-1.32)	0.614

AHI, apnea hypopnea index; BMI, body mass index; 95% CI, 95% confidence interval; CVD, cardiovascular disease; HR, hazard ratio; T90, percent time below oxygen desaturation 90%.

* Adjusted by age, sex, race, BMI, smoking status, alcohol use, diabetes mellitus, hypertension, history of major CVD, history of chronic respiratory disease, lipid-lowering medication use, benzodiazepine use, AHI and T90

Table S2. Sleep latency, wake after sleep onset and arousal index in self-reported and objective long sleep duration (> 8 hours)

	Objective SD (n=54)	Weekday SD (n=986)	Weekend SD (n=1602)
SL	2.1±4.2	13.7±21.5*	13.4±20.2*
WASO	26.1±10.5	75.1±50.0*	66.1±46.5*
ArI	14.3±7.2	20.7±11.6*	19.6±11.0*

ArI, arousal index; SD, sleep duration; SL, sleep latency; WASO, wake after sleep onset.

* $p<0.05$, compared with objective SD

Table S3. HRs and 95% CIs for WASO, SL and ArI associated with all-cause and CVD mortality

	Univariate model		Multivariable adjusted*	
	**HR (95% CI)**	**P**	**HR (95% CI)**	**P**
**All-cause mortality**				
WASO*	1.04 (1.03-1.05)	<0.001	1.02 (1.01-1.03)	<0.001
SL*	1.02 (1.01-1.04)	0.001	1.02 (1.01-1.03)	0.009
ArI*	1.09 (1.06-1.11)	<0.001	1.02 (0.99-1.05)	0.201
**CVD mortality**				
WASO*	1.04 (1.03-1.05)	<0.001	1.02 (1.01-1.03)	<0.001
SL*	1.01 (0.99-1.04)	0.341	1.01 (0.98-1.03)	0.692
ArI*	1.08 (1.03-1.13)	0.001	1.02 (0.96-1.07)	0.549

AHI, apnea hypopnea index; ArI, arousal index; BMI, body mass index; CHF, congestive heart failure; 95% CI, 95% confidence interval; COPD, chronic obstructive pulmonary disease; CVD, cardiovascular disease; HR, hazard ratio; MI, myocardial infarction; SL, sleep latency; T90, percent time below oxygen desaturation 90%; WASO, wake after sleep onset.

* per 5-unit increased

Each individual sleep variable including WASO, SL and ArI was adjusted by age, sex, race, BMI, smoking status, alcohol use, diabetes mellitus, hypertension, history of major CVD (MI, CHF and stroke), history of chronic respiratory disease (COPD, chronic bronchitis), lipid-lowering medication use, benzodiazepine use, AHI and T90

Figure S1. Correlation between objective sleep duration and self-reported sleep duration

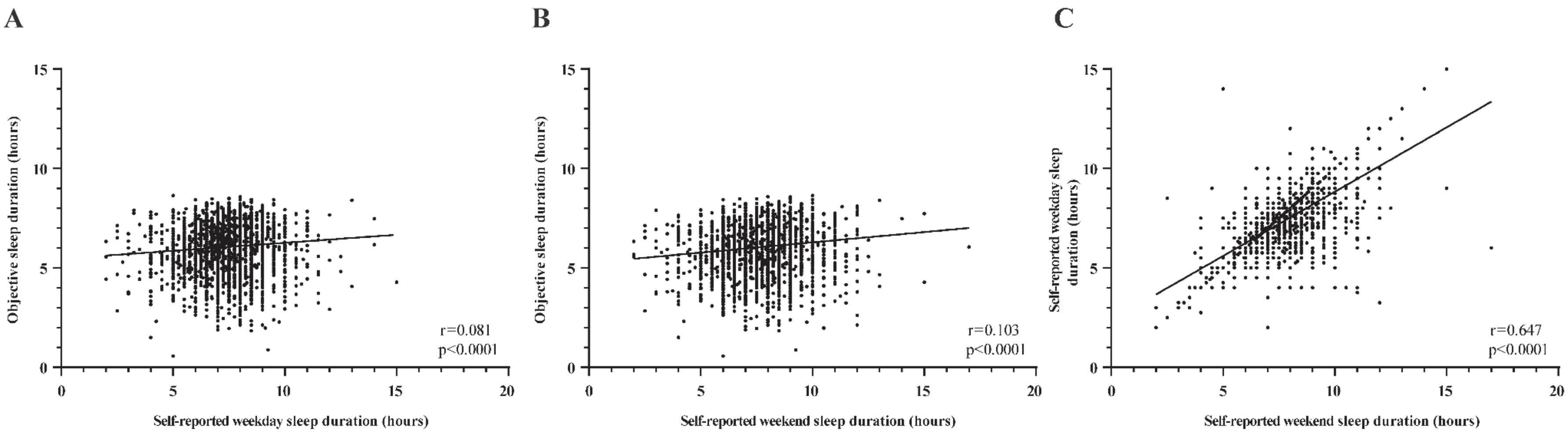

A. objective sleep duration and self-reported sleep duration on weekdays; **B.** objective sleep duration and self-reported sleep duration on weekends; **C.** self-reported sleep duration on weekdays and self-reported sleep duration on weekends.

Figure S2. Multivariable Cox proportional hazard ratio for all-cause mortality based on restricted cubic spline analysis of sleep duration in men and women

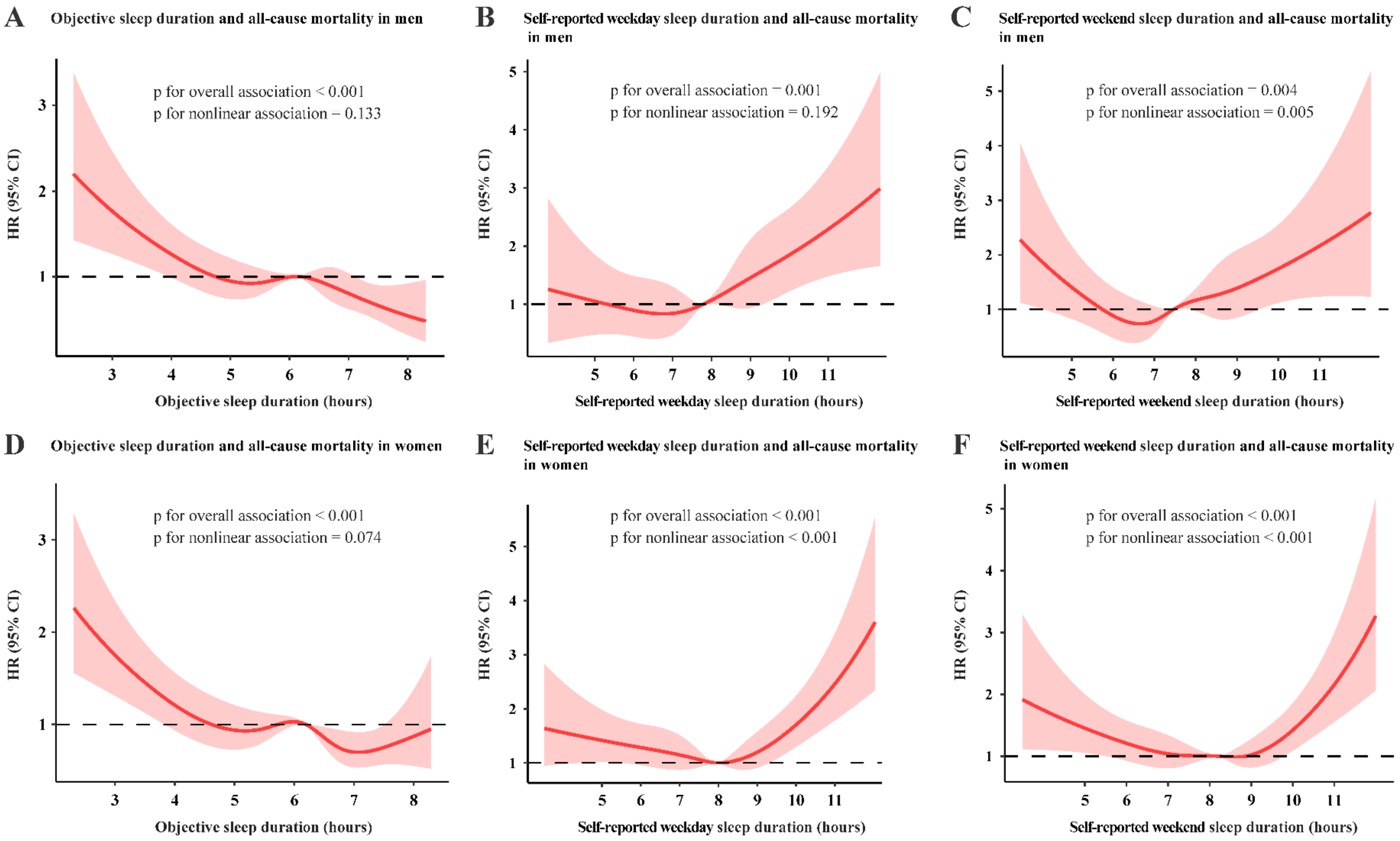

A. objective sleep duration and all-cause mortality in men; **B.** self-reported sleep duration on weekdays and all-cause mortality in men; **C.** self-reported sleep duration on weekends and all-cause mortality in men; **D.** objective sleep duration and all-cause mortality in women; **E.** self-reported sleep duration on weekdays and all-cause mortality in women; **F.** self-reported sleep duration on weekends and all-cause mortality in women.

CI, confidence interval; HR, hazard ratio.

Figure S3. Multivariable Cox proportional hazard ratio for all-cause mortality based on restricted cubic spline analysis of sleep duration in men and women

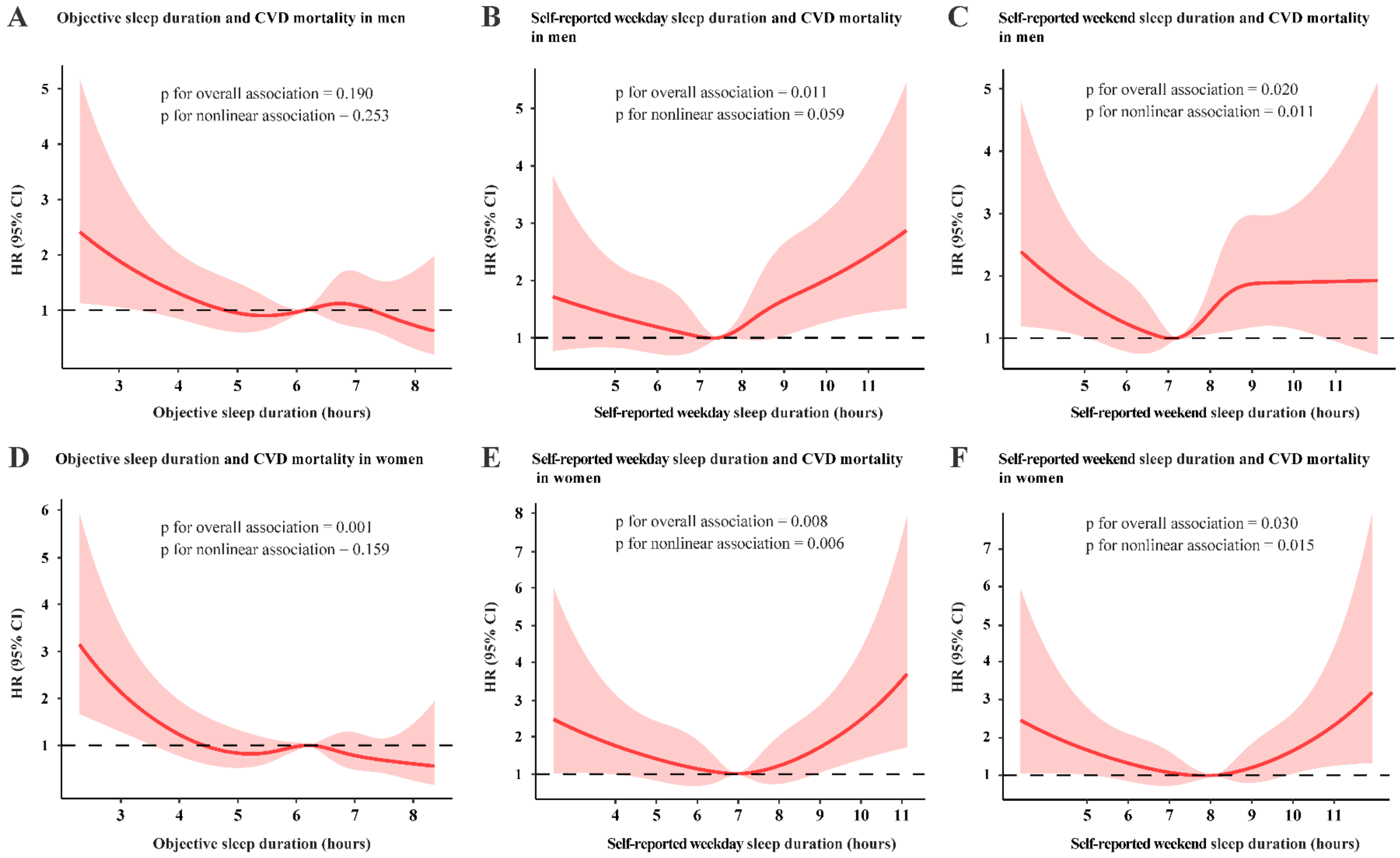

A. objective sleep duration and cardiovascular disease (CVD) mortality in men; **B.** self-reported sleep duration on weekdays and CVD mortality in men; **C.** self-reported sleep duration on weekends and CVD mortality in men; **D.** objective sleep duration and CVD mortality in women; **E.** self-reported sleep duration on weekdays and CVD mortality in women; **F.** self-reported sleep duration on weekends and CVD mortality in women.

CI, confidence interval; HR, hazard ratio.
